# Engineering catechol 1, 2-dioxygenase by design for improving the performance of the *cis, cis*-muconic acid synthetic pathway in *Escherichia coli*

**DOI:** 10.1038/srep13435

**Published:** 2015-08-26

**Authors:** Li Han, Pi Liu, Jixue Sun, Yuanqing Wu, Yuanyuan Zhang, Wujiu Chen, Jianping Lin, Qinhong Wang, Yanhe Ma

**Affiliations:** 1 Key Laboratory of Systems Microbial Biotechnology, Tianjin Institute of Industrial Biotechnology, Chinese Academy of Sciences, Tianjin 300308, China; 2 State Key Laboratory of Medicinal Chemical Biology and College of Pharmacy, Nankai University, Tianjin 300071, China

## Abstract

Regulating and ameliorating enzyme expression and activity greatly affects the performance of a given synthetic pathway. In this study, a new synthetic pathway for *cis, cis*-muconic acid (*cc*MA) production was reconstructed without exogenous induction by regulating the constitutive expression of the important enzyme catechol 1,2-dioxygenase (CatA). Next, new CatAs with significantly improved activities were developed to enhance *cc*MA production using structure-assisted protein design. Nine mutations were designed, simulated and constructed based on the analysis of the CatA crystal structure. These results showed that mutations at Gly72, Leu73 and/or Pro76 in CatA could improve enzyme activity, and the activity of the most effective mutant was 10-fold greater than that of the wild-type CatA from *Acinetobacter* sp. ADP1. The most productive synthetic pathway with a mutated CatA increased the titer of *cc*MA by more than 25%. Molecular dynamic simulation results showed that enlarging the entrance of the substrate-binding pocket in the mutants contributed to their increased enzyme activities and thus improved the performance of the synthetic pathway.

The microbial production of platform chemicals, which can be either directly used or further processed for the production of industrial-scale and high value-added products in the chemical industry, has recently gained tremendous interest and attention, especially with recent rapid advances in synthetic biology and metabolic engineering^1^,^2^,^3^. Adipic Acid, six-carbon dicarboxylic acid, is linked almost 90% to nylon production, and can be further processed into fibers for the production of carpeting, automobile tire cords and clothing^4^. Most commercial adipic acid is produced from cyclohexane through a two-step oxidation process. This inferior process exhibits a poor yield, and the chemicals used are environmentally harmful^5^. Alternatively, adipic acid can be produced from *cis, cis*-muconic acid (*cc*MA) by chemical hydrogenation. The production of *cc*MA from renewable resources by fermentation, followed by hydrogenation to adipic acid, would be desirable because such a route to adipic acid would be more environmentally friendly than the traditional petrochemical route^6^. Moreover, *cc*MA possesses reactive carboxylate groups and a conjugated double bond configuration that can be useful as a raw material for new functional resins, pharmaceuticals and agrochemicals^7^.

*cc*MA is not endogenously produced from renewable sugars by any known organisms, but it can be produced via the catabolism of aromatic compounds, such as phenol, toluene, benzyl alcohol and benzoic acid, by many organisms, including yeast, such as *Candida* sp., and bacteria, such as *Acinetobacter* sp., *Pseudomonas* sp., *Rhodococcus* sp., and *Sphingobacterium* sp.^7^,^8^,^9^. However, *cc*MA can also be produced from renewable glucose via the introduction of heterologous synthetic pathways that draw from naturally occurring intermediates in the shikimate pathway (Fig. 1)^5^,^10^,^11^,^12^,^13^,^14^. In all of these *cc*MA synthetic pathways, as well as the catabolic pathways of aromatic compounds, catechol 1,2-dioxygenase (CatA) is the last and most important enzyme for converting catechol to produce *cc*MA (Fig. 1). To improve the production of *cc*MA, it is necessary to obtain a CatA with higher activity.

The comprehensive substrate diversity and catalytic properties of many CatAs from *Acinetobacter, Alcaligenes*, *Arthrobacter*, *Geobacillus*, *Pseudomonas, Rhodococcus, Sphingomonas, Stenotrophomonas* and *Trichosporon* have been reported and described^15^,^16^. Additionally, global microbial genome and environmental metagenome sequencing efforts are also contributing ever-increasing genetic information on *catA*. These studies facilitate screening efforts to find CatAs with higher activities, which are needed for the efficient production of *cc*MA^15^,^17^. Moreover, many CatAs have been structurally elucidated using crystal structures and modeling studies^18^,^19^,^20^,^21^. The crystal structure of catechol 1,2-dioxygenase is formed from an αα, αβ and ββ dimer by two identical or non-identical subunits. A non-heme Fe(III) ion is incorporated into the catalytic center and ligated by two histidine residues, two tyrosine residues, and a hydroxyl ion in a trigonal-bipyramidal geometry^21^. The interactions of the enzyme with the substrate catechol to form a binding pocket involve Leu73, Pro76, Ile105, Pro108, Leu109, Arg221, Phe253 and Ala254 in *Acinetobacter* sp. ADP1, in which Ile105, Pro108, Phe253, and Arg221 are conserved in the catechol 1,2-dioxygenase family^18^,^19^,^20^,^21^,^22^. This structural information lays a solid foundation for the rational redesign of CatA to improve its catalytic activities and other related functions. Recently, the catalytic properties of CatA from *A. radioresistens* LMG S-13 were fine-tuned via the tailoring of a pocket shape to improving catalysis at the active site^22^. The mutation of residue Leu69 changed the substrate specificity, and some variants of Ala72 enhanced the *k*_*cat*_ toward chlorinated substrates. Although an increasing number of proteins have been engineered for altered substrate specificity/selectivity by ration design, the engineering of highly efficient enzymatic pathways for industrial-scale fuel and chemical production by increasing the catalytic activity of the key enzymes remains an overwhelming challenge and requires expanded efforts in metabolic engineering and synthetic biology^23^.

In this study, a *cc*MA synthetic pathway in *Escherichia coli* was reengineered to produce *cc*MA without exogenous induction by regulating the constitutive expression of CatA. Next, new mutants derived from wild-type *Acinetobacter* sp. ADP1 CatA (WT) were designed and constructed to enhance enzyme activity by reshaping the substrate-binding pocket. Successful mutants increased the titer of *cc*MA in *E. coli*. The biochemical studies and computational modeling of the mutated CatAs further elucidated the underlying molecular mechanism of the improved enzymatic activities. Our results show that rational redesign can enable the improved performance of key enzymes and synthetic pathways and can be applied to many other metabolic engineering and synthetic biology studies.

## Results

### Reconstructing the *cc*MA synthetic pathway by constitutive promoter engineering

At present, the expression of the catechol 1,2-dioxygenase gene (*catA*) for *cc*MA production in *E. coli* is typically under the control of the lac promoter and induced by exogenous isopropyl-α-D-thiogalactopyranoside (IPTG)^5^,^10^,^11^,^14^. Thus, the utility of an inducible promoter-based *cc*MA synthetic pathway is limited in future industrial applications. Therefore, we investigated the possibility of replacing the lac promoter with constitutive promoters. Three constitutive promoters from the antibiotic cassettes of pACYC177 or pACYC184, P_kan_, P_cm_, and P_tc_, as well as PL25 from our synthetic promoter library^24^, were used to replace the lac promoter of the catechol 1,2-dioxygenase gene (Fig. S1A).

The constitutive promoter-*catA* expression cassettes were constructed by either fusion PCR or chemical synthesis and then used to replace the inducible-*catA* expression cassette in pKD8.292^5^ to obtain four recombinant plasmids, pKD8.292K, pKD8.292C, pKD8.292T and pKD8.292PL25, with constitutive promoters P_kan_, P_cm_, P_tc_, and PL25, respectively (Fig. S1B). Then, pKD8.292K, pKD8.292C, pKD8.292T and pKD8.292PL25 were each co-transformed with the plasmid pKD8.243^5^ containing 3-dehydroshikimate dehydratase (*aroZ*) and protocatechuate decarboxylase (*aroY*) into *E. coli* AB2834 to obtain new engineered strains (*E. coli* WZK, WZC, WZT, and WZPL25, respectively) with *cc*MA heterologous synthetic pathways. All strains with constitutive promoters produced different amounts of *cc*MA without IPTG induction (Table 1), and the strain with the synthetic promoter PL25 (*E. coli* WZPL25) exhibited a similar titer as that of the strain with the inducible lac promoter (*E. coli* WZI). These results indicate that it is possible to replace the inducible promoter with a suitable constitutive promoter. However, these reconstructed *cc*MA synthetic pathways still accumulated the metabolic intermediate catechol (Table 1). This result implied that it would be necessary to increase the enzymatic activity of CatA.

### Prediction and analysis of the structure-based CatA redesign to increase enzyme activity

Based on the crystal structure of CatA from *Acinetobacter* sp. ADP1^21^, as well as data from the Protein Data Bank (PDB ID: 1DLT), the substrate-binding pocket model of wild-type CatA was reconstructed using PyMOL (Fig. 2). As shown in this substrate-binding pocket model, residues 105–109 (purple loop) above the substrate catechol, residues 199–203 and 218–221 below the substrate (red parallel loop), and residues 253–256 beside the substrate (green loop) are critical to maintain the shape of the binding pocket, and we hypothesized that introducing any mutation would likely seriously lower or destroy the enzymatic activity of CatA. However, residues 69–78 (blue helix) to the upper left of the substrate assist in regulating the size of the substrate-binding pocket. At this location, these residues act as a cap on the edge of the binding pocket, making these residues a suitable choice for mutation to improve enzymatic activity. Thus, two design strategies were considered for changing enzyme activity. The first strategy was to increase the substrate-enzyme binding energy, and the second strategy was to enlarge the binding cavity to increase substrate access.

As shown in Fig. 2, Gly72 is located immediately above the substrate and is one of the key residues interacting with the substrate catechol. We posited that if Gly72 is mutated to Ala or Val to increase hydrophobicity, this mutation might influence interactions with catechol, as well as the location of Pro108, to change the size of the pocket. Additionally, the interaction of Pro108 with hydrophobic residues at position 72 might twist the blue helix slightly. Thus, Leu73 might yield more space for a substrate to enter the binding pocket. Moreover, the larger branched chain of Val72 may produce a bigger cavity. Leu73 directly controls the size of the pocket like a switch. If it is mutated to Phe or Met, these hydrophobic amino acid residues might push away Cys202, thus enlarging the binding-pocket entrance while maintaining hydrophobicity. However, if the extended side chains of Phe and Met point toward the substrate after mutation, they might also block entry into the binding pocket.

Similar to Gly72, we considered the mutation of Pro76 to Ala, Gly or Val. Based on our structural predictions, the mutation of Pro76 might lead to two outcomes, either altering the hydrophobic interactions with the substrate catechol or giving structural flexibility to the blue helix. If Pro76 is mutated into Ala76 or Gly76, these changes may decrease hydrophobic interactions with the substrate catechol and Ile105 and result in enlargement of the binding pocket. If Pro76 is mutated to Val76, this mutation may increase hydrophobic interactions with the substrate but may also push away Ile105 farther. Thus, this mutation might also enlarge the binding pocket. Furthermore, the mutation of Pro76 to Ala76 can bring flexibility to the blue helix, thus bringing flexibility to the direction of Leu73. This change will enlarge the entrance of the binding pocket. In addition, double mutations at residues Leu73 and Pro76 may have synergistic effects on the size of the binding pocket. Based on the predictions and analyses above, eight single mutations and one double mutation were designed for experimental validation (Table 2).

### Experimental validation of the structure-based redesign of CatA for increasing enzyme activity and improving the production of *cc*MA

To verify our structure-based functional predictions and efficiently screen the mutated CatAs, site-specific mutagenesis was used to generate the mutants G72P, G72A, G72V, L73F, L73M, P76A, P76G, P76V and L73F/P76A with the recombinant plasmid pKD8.29PL25. pKD8.29PL25 derivatives with different mutations were co-transformed with pKD8.243 containing the genes *aroZ* and *aroY* into *E. coli* AB2834 to obtain engineered *E. coli* strains for the analysis of CatA enzymatic activity, as well as for the improved production of *cc*MA. In *E. coli* AB2834, *aroE* was mutated to redistribute the flux and thus improve the production of *cc*MA^6^.

Crude cell extracts from the engineered *E. coli* strains with wild-type or mutated CatAs exhibited different specific activities of catechol 1,2-dioxygenase (Table 2). Four variants, G72A, L73F, P76A, and L73F/P76A, showed higher specific activities, and the mutant P76A had the highest specific activity with more than 10-fold that of the WT toward the substrate catechol. Based on our previous modeling and mutation designs, the behavior of nearly half of the mutations were consistent with our prediction analysis. The successful rate for predictions was not comparatively high, but the proposed hypothesis for improving enzymatic activity via enlarging the binding pocket was still helpful. In addition, engineering the substrate specificity of CatA was reported in the previous report by tailoring the catalysis at the active sites^22^, but improving the enzymatic activity of CatA significantly by structural-based redesign was first reported here.

To test the influence of the mutated CatAs on the performance of the *cc*MA synthetic pathway in *E. coli*, the production of *cc*MA and certain related metabolic intermediates were measured during growth in a minimal medium. As shown in Fig. 3A, the strains harboring mutated CatAs with higher enzymatic activities showed increased *cc*MA production. Although the G72A strain did not show a significant difference versus WT, the other three mutants, L73F, P76A and L73F/P76A, led to significantly increased production of *cc*MA. The strain with mutation P76A produced the highest amount of *cc*MA, 1.53 g/L, an approximately 26.4% improvement compared with the strain with WT CatA, whereas L73F and L73F/P76A produced approximately 1.42 g/L (a 17.4% improvement) and 1.48 g/L *cc*MA (a 22.3% improvement), respectively.

**Figure 1:**
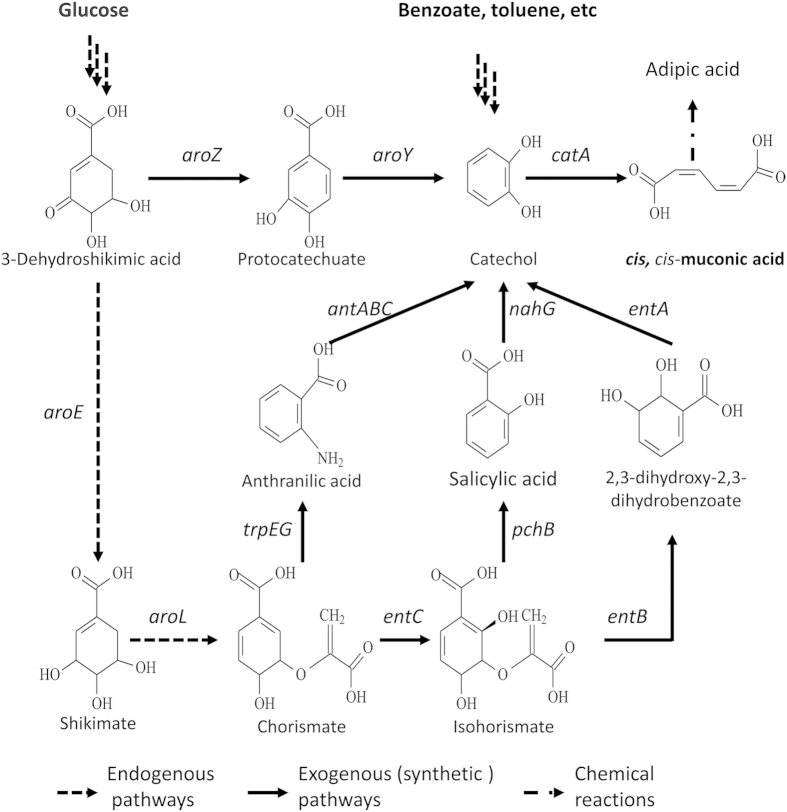
Summary of synthetic pathways for the production of *cis, cis*-muconic acid from glucose. *aroZ* encodes 3-dehydroshikimate dehydratase; *aroY* encodes protocatechuate decarboxylase; *catA* encodes catechol 1,2-dioxygenase; *trpEG* encodes anthranilate synthase; *antABC* encodes anthranilate 1,2-dioxygenase; *entC* encodes isochorismate synthase; *pchB* encodes isochorismate pyruvate lyase; *nahG* encodes salicylate 1-monoxygenase; *entB* encodes isochorismatase; *entA* encodes 2,3-dihydro-2,3-DHBA dehydrogenase.

**Figure 2:**
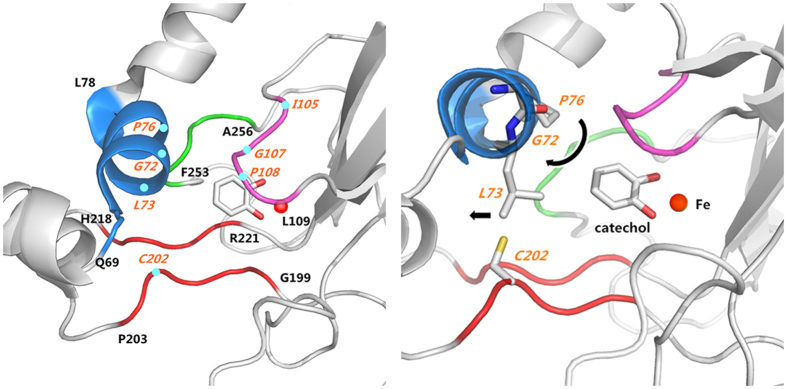
Substrate-binding pocket model of CatA *Acinetobacter* sp. ADP1 with different viewpoints. The blue helix, purple loop, green loop and red parallel loop are the key structures that determine pocket volume. G72 interacts with P108, and P76 interacts with I105. Both of these residues have interactions with the substrate (Left panel). The mutation of P76 and G72 might change the dihedral angles ϕ and ψ (Black arrows), thus twisting the blue helix and moving L73 to yield a wider entrance (Right panel).

**Figure 3:**
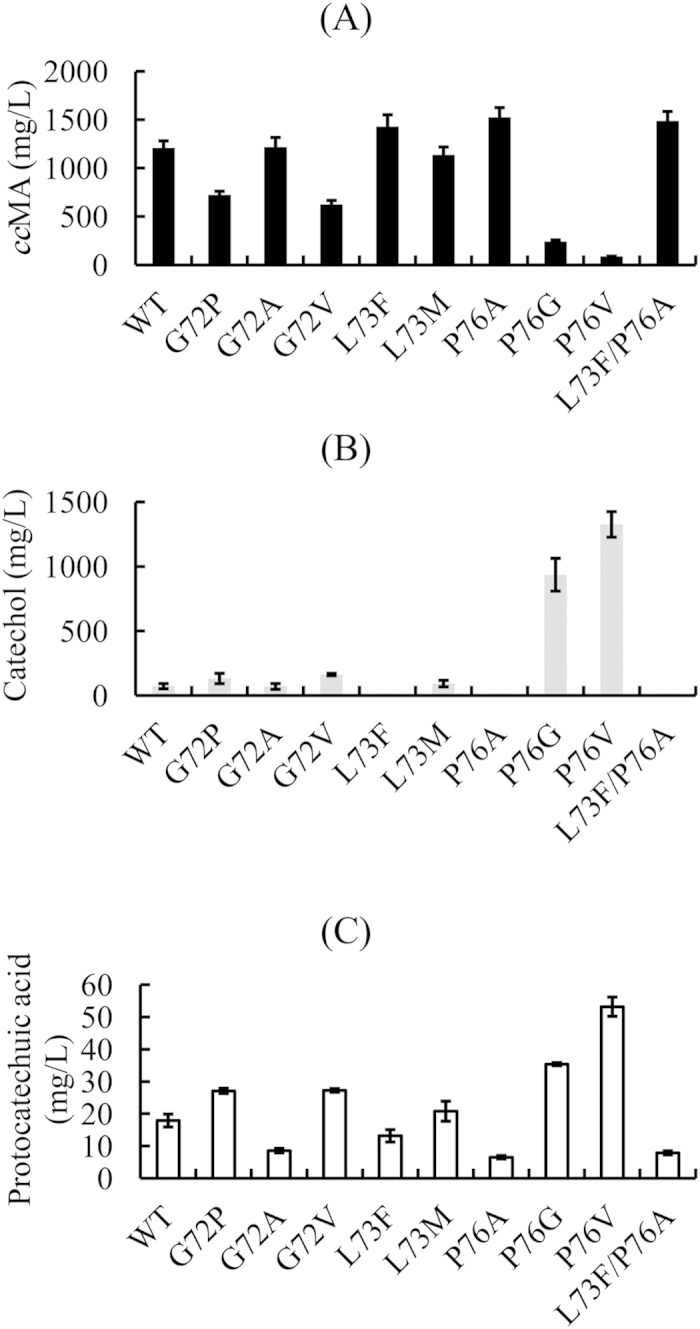
*cc*MA, catechol and protocatechuic acid production in engineered *E. coli* strains with wild-type or mutated CatAs.

Catechol accumulated in the medium when the enzymatic activity of CatA decreased (Fig. 3B). As shown in Fig. 3C, increased CatA activity also contributed to a decrease in the intermediate protocatechuic acid. Our results indicate that the performance of the *cc*MA synthetic pathway may be improved by redesigning key enzymes. However, some protocatechuic acid still accumulates in our engineered strains. This finding demonstrates that the activity of AroY must be further increased (Fig. 1). Although we could largely increase the enzymatic activity of CatA, the titer of *cc*MA from the engineered *E. coli* with mutated CatAs was not increased dramatically. The supply of precursors in the *cc*MA synthetic pathway was likely not adequate, thus limiting the increase of the *cc*MA titer. This hypothesis was confirmed by our ongoing engineering studies for improving the production of 3-dehydroshikimic acid (Fig. 1), and subsequently increasing ccMA production (unpublished data).

### Biochemical studies and computational simulation to elucidate the mechanisms of the effective mutants

**Figure 4:**
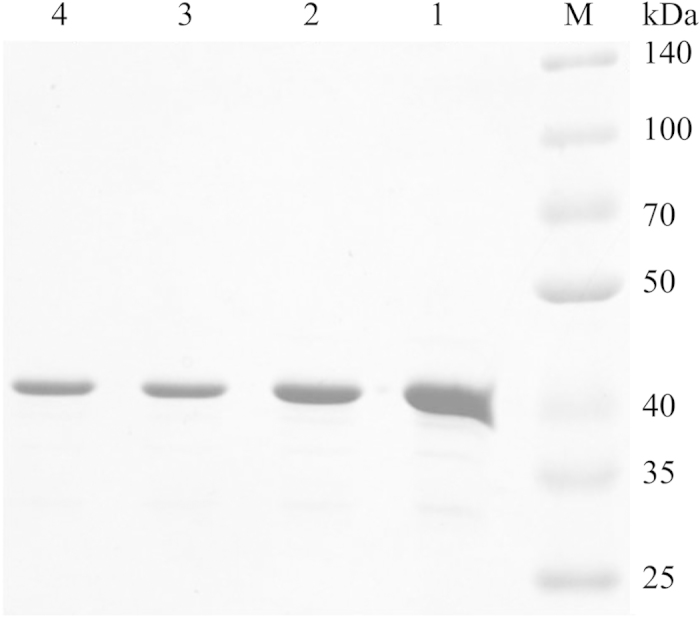
SDS-PAGE analysis of purified wild-type and mutant CatA proteins. Lane M, protein marker; lane 1, WT; lane 2, L73F; lane 3, P76A; lane 4, L73F/P76A.

To investigate how the mutation of CatA affected enzymatic activity and the production of *cc*MA, biochemical studies of wild-type and mutated CatAs were first conducted to elucidate the underlying functions. Based on previous results, three variants, L73F, P76A and L73F/P76A, as well as WT were expressed and purified from *E. coli* for biochemical characterization (Fig. 4). *K*_*m*_ and *k*_*cat*_ values of the recombinant proteins were measured and calculated based on the Michaelis-Menten equation by nonlinear fitting (Table 3). The *K*_*m*_ values of L73F and P76A were nearly 20- and 9-fold higher than that of WT, respectively. This result indicates that the residues at positions 73 and 76 greatly influence substrate binding. However, the *k*_*cat*_ of P76A and L73F/P76A increased by more than 5-fold versus WT, emphasizing their increased catalytic activities and resulting in increased *cc*MA production. The L73F mutation exhibited a clear effect on *K*_*m*_, but its *k*_*cat*_ value was similar to that of WT. Hence, the production of *cc*MA by the L73F-containing strain was less than that of P76A or L73F/P76A.

To further investigate how the L73F, P76A and L73FP76A mutations affect the catalytic activity of CatA and *cc*MA production, molecular dynamic (MD) simulations were used to elucidate the underlying mechanisms. Based on the previous structural analysis^20^, a non-heme Fe(III) ion is a cofactor in the binding pocket of CatA, and this non-heme Fe(III) ion plays a key role in maintaining enzyme activity. When the substrate catechol enters, the metal should be ligated by five ligands: catechol, two histidine residues, one tyrosine residue and a water molecule, leading to the formation of a hexacoordinated octahedron-shaped structure^19^. To better analyze the molecular mechanism of CatA, an iron force field (Fig. S2) was constructed using Pang’s method^25^. With this iron force field, wild-type CatA and the three mutants L73F, P76A and L73F/P76A were subjected to 2-ns MD simulations to verify the experimental results.

**Figure 5:**
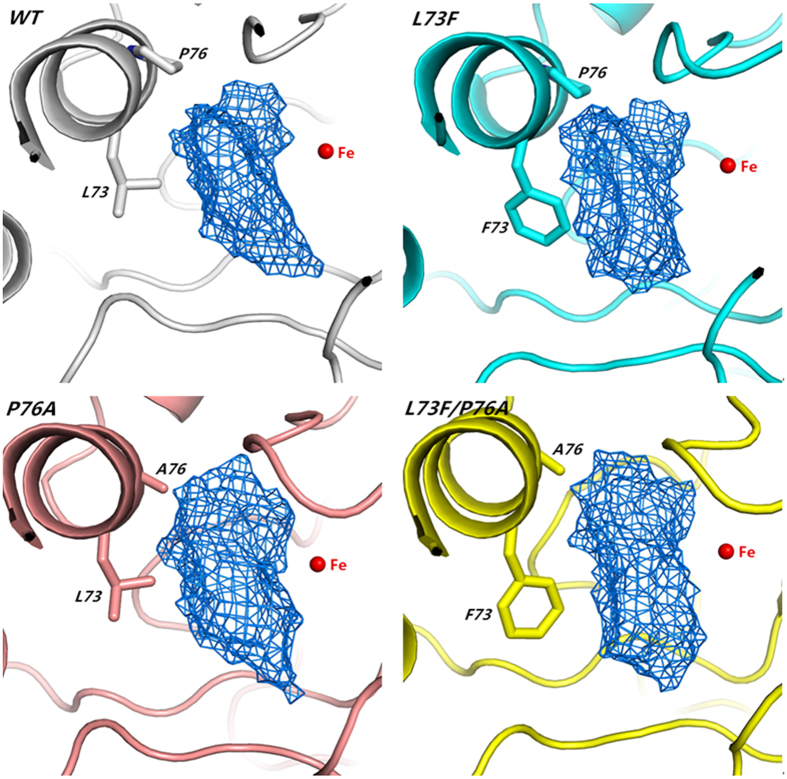
Pocket volume of wild-type and mutated CatA. The blue lattice displays the volume of the binding-pocket cavity. The pocket volumes of WT, L73F, P76A and L73F/P76A are 132, 123, 178 and 152 Å^3^, respectively.

Because the distances between residues 73 and Cys202 or Leu109 and between residues 76 and Ile105 or Gly107 will decide the volume of the binding pocket, these distances were measured in WT and the three mutants based on the simulation results (Table 4, Figs S3 and S4). As shown in Table 4, the mutation of Leu73 to Phe decreased the distance between residues 73 and Leu109 or Cys202. However, the mutation of Pro76 to Ala increased the distance between residues 76 and Ile105 or Gly107. The double mutation L73F/P76A exhibited a similar distance to Leu109 or Cys202 and an increased distance to Ile105 or Gly107 compared with those in WT. The distance changes should affect the volume of the binding pocket. Therefore, the pocket volumes of the WT and mutants were simulated and measured by POVME^26^. As shown in Table 4 and Fig. 5, the pocket volumes of WT, L73F, P76A and L73F/P76A were 132, 123, 178 and 152 Å^3^, respectively. The shortened distance between Phe73 and Leu109 narrowed the width of the pocket gate, but the cavity volume in L73F did not change considerably. This result explains why the *k*_*cat*_ value of L73F is lower than that of WT. For P76A, Gly107 pushed Ala76 away when Pro76 was mutated to Ala, and this resulted in an expanded cavity and pocket volume. With the combinatorial effect of L73F and P76A, the pocket volume of the double mutant L73F/P76A was smaller than that of P76A but larger than that of L73F. The volumes of the mutants corresponded with their *k*_*cat*_ values. Our results showed that the computational modeling was in good agreement with the experimental outcome, and increasing the volume of the binding pocket will facilitate substrate entry or product release, thus enhancing the catalytic rate of the enzyme. This finding implies that the binding pocket is a possible target for improving the catalytic efficiency of any enzyme.

## Discussion

As a high value-added bioproduct with reactive dicarboxylic groups and conjugated double bonds, *cc*MA has gained increasing interest due to its potential applications in the manufacture of commercially important bulk chemicals, such as adipic acid, terephthalic acid and trimellitic acid, new functional resins, bioplastics, food additives, agrochemicals, and pharmaceuticals^27^. Although many microorganisms can convert some aromatic compounds, such as catechol, benzoate and toluene, to *cc*MA, it is almost impossible to develop an economically feasible and environmentally friendly process based on these bioconversions. Hence, recent efforts have focused on a promising approach for more sustainable production of *cc*MA based on the design of a *de novo* biosynthetic pathway (Fig. 1). As a result, this compound was produced by engineered *E. coli* or yeast with biomass-derived glucose as the substrate, although the performances of the majority of these synthetic pathways must be further improved for future industrial applications.

As shown in Fig. 1, all synthetic pathways for *cc*MA production utilize the same last biochemical reaction catalyzed by CatA. Therefore, the selection of a CatA with suitable protein expression and activity is key for improving the performance of the synthetic pathways^15^,^17^. Unfortunately, the expression of catechol 1,2-dioxygenase in many reported *cc*MA synthetic pathways used the lac-derived expression system^5^,^10^,^11^,^14^. This protein expression system requires an inducer in the medium, the un-metabolizable compound IPTG. IPTG is expensive and cannot be used on an industrial scale. It is highly toxic, and it yields a highly intense response. Hence, in this study, we engineered the expression of CatA by a constitutive promoter instead. According to our results (Table 1), the selected synthetic promoter PL25 performed similarly to, even better than the inducible lac promoter. This genetic modification will contribute to future industrial applications. Moreover, this selected synthetic promoter PL25 was from our designed promoter library with different strengths, which would be very useful to regulating other synthetic pathways constitutively^24^.

Enhancing the activity or catalytic efficiency of key enzymes by protein redesign is another important strategy for improving the performance of synthetic pathways^23^,^28^. Many proteins can be engineered for altered substrate specificity/selectivity, increased catalytic activity, reduced mass-transfer limitations through specific protein colocalization, and reduced substrate/product inhibition. There are many reports of engineered enzymes that increase the overall performance of a pathway. Previous studies have demonstrated that substrate specificity/selectivity can be improved by reshaping or enlarging the binding pocket of an enzyme^22^,^29^; however, in this study, we demonstrated that enlarging the binding pocket could also enhance enzymatic activity (Table 4 and Fig. 5), thus improving the performance of our *cc*MA synthetic pathway.

Based on computational modeling, we speculated that the larger binding pocket facilitates substrate entry or product release, resulting in increased catalytic activity. Enlarging the substrate-binding pocket of an enzyme would make it have ability to catalyze the bigger substrate. However, for the smaller substrate or original substrate, enlarging the binding pocket would weak the interaction energy. As a consequence, the *K*_*m*_ of the enzyme would increase. On the other hand, the bigger binding pocket would give substrate more chance to enter the pocket. Therefore, it would also increase *k*_*cat*_. In this study, we try to enlarge the entrance of binding pocket to increase *k*_*cat*_ while maintaining the volume of pocket bottom to prevent *K*_*m*_ from increasing dramatically (Table 3). This strategy should also be applied to other protein redesigns for increasing enzymatic activity and improving the performance of metabolic pathways.

In addition, in this study, we did dramatically improve the enzymatic activity of CatA by structure-based rational design, but the improvement of *cc*MA production for the strain with effective mutated CatA was not so significant. This confirmed the fact that decreasing one specific metabolic bottleneck will automatically make another one to rise. Hence, further engineering other enzymes for new limited steps with similar redesign strategy presented in this study is very necessary to further improve the production of *cc*MA.

## Methods

### Bacterial strains and media

*E. coli* trans5a from Beijing Transgen Biotech (Beijing, China) was used for plasmid construction and molecular cloning. *E. coli* AB2834 with a 3-dehydroshikimate dehydrogenase (AroE) deficiency (from The Coli Genetic Stock Center at Yale University) was used for *cc*MA production. *E. coli* BL21(DE3) was used for protein expression to obtain the purified recombinant proteins for kinetic analysis. All strains used in this study are shown in Table S1 (Supplementary data). LB medium containing 10 g/L tryptone, 5 g/L yeast extract, and 10 g/L NaCl was used for cell growth. Modified M9 minimal medium containing 40 mg/L shikimate, 10 g/L glucose, 6 g/L Na_2_HPO_4_, 0.5 g/L NaCl, 3 g/L KH_2_PO_4_, 2 g/L NH_4_Cl, 246.5 mg/L mg/L MgSO_4_, and 14.7 mg/L CaCl_2_ was used for *cc*MA production. If necessary, chloramphenicol (Cm^R^), spectinomycin (Spe^R^), kanamycin (Kan^R^), or IPTG was added to the medium at 34 μg/mL, 50 μg/mL, 50 μg/mL, or 1 mM, respectively.

### Plasmid construction

The plasmid pKD8.243 was obtained from *E. coli* ATCC 69875^5^. The primers used for plasmid construction in this study are listed in Table S2, and the restriction enzyme sites are underlined in the primers. The 220-bp constitutive promoters P_kan_, P_cm_, and P_tc_ from the Kan^R^, Cm^R^, and Tc^R^ cassettes in pACYC177 or pACYC184 were amplified by polymerase chain reaction (PCR) with the primers Pkan-EcoRI-5/Pkan-3, Pcm-EcoRI-5/Pcm-3, and Ptc-EcoRI-5/Ptc-3, respectively. The 1080-bp *catA* gene for each constitutive promoter cassette was amplified from pKD8.292^5^ by PCR with the primers CatA-kan-5/CatA-EcoRI-3, CatA-cm-5/CatA-EcoRI-3, or CatA-tc-5/CatA-EcoRI-3, respectively. Then, the PCR products of each constitutive promoter and the *catA* gene were ligated by fusion PCR using the overlapping sequences that were introduced by each primer pair. After *Eco*RI digestion, the 1.3-kb fusion PCR products were each ligated with the 4.2-kb *Eco*RI- and calf intestinal alkaline phosphatase-digested pKD8.292 product to obtain the new recombinant plasmids (Fig. S1B). In pKD8.292K, pKD8.292C and pKD8.292T, the lac promoters are replaced by the constitutive promoters from the Kan^R^, Cm^R^, or Tc^R^ cassettes (Fig. S1A), respectively. Wild-type *catA* from *Acinetobacter* sp. ADP1^5^ with the constitutive synthetic promoter PL25 (TCTAGAATATGTTATCTCTGGCGGTGTTGACAACGAGCTGGACAACTGGTATAATGCCACATGAGCGGATAACAATTTCAAGGAGGACAGCTCCATGG)^24^ was chemically synthesized by GENEWIZ, Inc. in Suzhou, China (Fig. S1C). This synthetic DNA fragment was cloned into pKD8.292 to obtain the recombinant plasmid pKD8.29PL25 by replacing the original *catA* with the inducible lac promoter via *Eco*RI restriction digestion and ligation. The recombinant plasmids with different mutated *catA*s were constructed by inverse PCR of pKD8.29PL25. All recombinant plasmids in this study were confirmed by DNA sequencing and are shown in Table S1.

### Microbial production of *cc*MA and metabolite analysis

Different plasmids with wild-type or mutated *catA*, as well as pKD8.243, were transformed into *E. coli* AB2834 to evaluate the production of *cc*MA. The engineered strains were inoculated in 3 mL of LB medium. Overnight cultures of these strains were diluted 1:10 into modified M9 minimal medium containing 10 g/L glucose, appropriate antibiotics and/or IPTG (for the lac promoter) and cultivated at 37 °C for 36 h with shaking at 220 rpm. From each culture, 1 mL was collected and centrifuged at 10,000 g to remove cells for metabolite analysis. Metabolites in the culture supernatants, including glucose, *cc*MA and other metabolites, were quantified by HPLC using a reverse-phase ZORBAX C18 (Agilent Technologies) or HPX-87H column (Bio-Rad Laboratories). All experiments were performed minimally in triplicate to ensure reproducibility, and the results represent the mean ± standard deviation.

### Expression, purification and enzyme assay of catechol 1,2-dioxygenase

Wild- type and mutant *catA* were cloned into the expression plasmid pET30a(+) between *Nde*I and *Xho*I to obtain recombinant plasmids (pET-CatAWT, pET-CatAL73F, pET-CatAP76A and pET-CatAL73F/P76A, Table S1) for protein overexpression to obtain the purified recombinant protein for kinetic assays. *E. coli* BL21(DE3) with the different recombinant plasmids (*E. coli* CatAWT, *E. coli* CatAL73F, *E. coli* CatAP76A and *E. coli* CatAL73F/P76A) were induced using 1 mM IPTG for 7 h at 20 °C in LB medium for protein production. For purification, the cells were harvested by centrifugation and disrupted by ultrasonication in 20 mM phosphate buffer (pH 7.4) containing 20 mM imidazole and 0.25 M NaCl. The supernatant with the recombinant protein was obtained by centrifugation at 10,000 g for 30 min, filtered through a 0.22-μm membrane and then purified by Ni^+^-affinity chromatography from GE Healthcare Life. Imidazole in the purified protein was removed by tube ultrafiltration. The resulting enzyme solution was used for activity assays. The reaction was performed in a 1 mL system containing 0.2 mM catechol, 100 μM potassium phosphate buffer (pH 7.4), and 100 μL of enzyme solution at 25 °C for 20 min. Enzyme activity was measured and calculated by the production of *cc*MA via HPLC analysis. The protein concentration was determined with the Thermo Scientific Pierce BCA quantification assay kit.

### Kinetic parameter determination

Enzyme catalytic parameters (Michaelis-Menten constant *K*_*m*_ and *k*_*cat*_) were calculated by measuring the initial linear rates of the enzymatic reaction after the addition of different concentrations of catechol ranging from 0 to 200 μM in 100 mM potassium phosphate buffer (pH 7.4). After the addition of the purified enzyme, the reactions were performed, and the OD_260_ was monitored at 25 °C using with Spectramax M^2e^ apparatus (Molecular Devices, USA). The extinction coefficient of the catechol oxidation product was determined as ε_260nm_ = 16,800 M^−1^ cm^−1^. Three independent measurements were performed. *K*_*m*_ and *k*_*cat*_ were calculated based on the Michaelis-Menten equation. One unit was defined as the amount of enzyme producing 1 μmol *cc*MA per minute.

### Computational modeling and simulations

The wild-type model of CatA from *Acinetobacter* sp. ADP1 was constructed using PyMOL (Version 1.5.0, Schrödinger LLC) based on the crystal structure of CatA (PDB: 1DLT)^21^. The mutant structural models were constructed based on the 1DLT PDB file by renaming the target residue, removing the line of the different atom name between the original residue and mutated residue, and using the leap program from Amber Tools 12 and Amber12^30^ to add the missing atoms. Finally, the entire system was optimized using 5,000 steps of minimization. The iron force field was constructed using Pang’s method^25^. MD simulation for the three mutants L73F, P76A, and L73F/P76A and WT was conducted using the Leap module of Amber12^30^,^31^ with the standard Amber99SB force field and manually creating an iron force field based on the crystal structure from the Protein Data Bank (PDB ID: 1DLT). All simulations were run with a 0.5-fs time step and gradually reduced constraints on protein and langevin dynamics^32^ for temperature control. Then, the final phase of equilibration of every system was used to run a 2 ns MD simulation. POVME was used to calculate the volume of the binding pocket^26^. Some detailed information on computational modeling and simulation are provided in the supplementary methods.

## Additional Information

**How to cite this article**: Han, L. *et al.* Engineering catechol 1, 2-dioxygenase by design for improving the performance of the *cis*, *cis*-muconic acid synthetic pathway in* Escherichia coli.* *Sci. Rep.* **5**, 13435; doi: 10.1038/srep13435 (2015).

## Supplementary Material

Supplementary Information

## Figures and Tables

**Table 1:** *cc*MA production by engineered strains with different constitutive promoters or an inducible promoter*.

Strains	*cc*MA (g/L)	Catechol (g/L)	Yield** (%)
*E. coli* WZK	0.34 ± 0.05	0.92 ± 0.08	3.9 ± 0.6
*E. coli* WZC	0.43 ± 0.02	0.85 ± 0.07	5.3 ± 0.3
*E. coli* WZT	0.71 ± 0.03	0.45 ± 0.03	9.3 ± 0.3
*E. coli* WZPL25	1.21 ± 0.07	0.07 ± 0.02	22.2 ± 0.5
*E coli* WZI*	1.15 ± 0.12	0.09 ± 0.02	20.1 ± 0.3

^*^his strain was a control. During fermentation, 1 mM IPTG was added to the fermentation medium to induce the expression of catechol 1,2-dioxygenase. Errors (standard deviations) were calculated based on at least three independent experiments.

^**^Yield: produced *cc*MA against consumed glucose.

**Table 2:** Specific activities of catechol 1,2-dioxygenase in crude cell extracts from different engineered strains with wild-type and mutated *catA.*

Strains*	Specific Activity (U/mg)**
WT	2.489 ± 0.112
G72P	0.006 ± 0.002
G72A	15.828 ± 0.491
G72V	0.004 ± 0.001
L73F	6.425 ± 0.301
L73M	0.0652 ± 0.003
P76A	25.2629 ± 0.626
P76G	0.043 ± 0.002
P76V	0.005 ± 0.002
L73F/P76A	14.019 ± 0.549

^*^WT indicates *E. coli* AB2834 containing pKD2.834A and pKD2.292 with wild-type *catA*. Other strains contain different mutated *catA*s.

^**^1U = 1 μmol *cc*MA produced per minute at 24 °C. Errors (standard deviations) were calculated based on at least three independent experiments.

**Table 3:** Enzymatic kinetic parameters of wild-type and mutated CatAs after purification against the substrate catechol*.

Enzymes	*K*_*m*_(μM)	*k*_*cat*_(S^−1^)
WT	7.88 ± 1.239	7.86 ± 0.248
L73F	151.09 ± 10.299	5.65 ± 0.163
P76A	65.91 ± 7.753	46.56 ± 0.201
L73F P76A	45.96 ± 7.691	39.50 ± 0.221

^*^WT indicates wild-type CatA. Others are mutated proteins. Errors (standard deviations) were calculated based on at least three independent experiments.

**Table 4:** Distances (Å) between certain key residues and the size of the active pocket for wild-type and mutated CatAs*.

Characteristics	L73F	P76A	L73F/P76F	WT
Leu73 or Phe73 to Leu109	6.7 ± 0.4	7.9 ± 0.4	7.6 ± 0.4	7.6 ± 0.4
Leu73 or Phe73 to Cys202	3.7 ± 0.4	4.3 ± 0.4	3.9 ± 0.4	3.9 ± 0.3
Pro76 or Ala76 to Ile105	4.1 ± 0.3	4.2 ± 0.3	4.7 ± 0.4	3.7 ± 0.3
Pro76 or Ala76 to Gly105	4.5 ± 0.3	6.7 ± 0.2	6.7 ± 0.2	5.1 ± 0.3
Size of active pocket (Å^3^)	123 ± 10	178 ± 13	152 ± 11	132 ± 12

^*^50 snapshots of last 250 ps trajectories (5 ps per snapshot) during molecular dynamic simulation were used to calculate the distance and the volume. The results represent the mean ± standard deviation.
