# Supplementary material for: Engineering catechol 1, 2-dioxygenase by design for improving the performance of the cis, cis-muconic acid synthetic pathway in Escherichia coli
Source: Sci Rep. 2015 Aug 26;5:13435. doi: 10.1038/srep13435 (PMC4549619; doi:10.1038/srep13435)
Supplement: Supplementary Information [file srep13435-s1.pdf]

Engineering catechol 1, 2-dioxygenase by design for improving the performance of the *cis, cis*-muconic acid synthetic pathway in *Escherichia coli*

Li Han*[1], Pi Liu*[2], Jixue Sun[2], Yuanqing Wu[1], Yuanyuan Zhang[1], Wujiu Chen[1], Jianping Lin[1, 2]#, Qinhong Wang[1]#, Yanhe Ma[1]

[1]Key Laboratory of Systems Microbial Biotechnology, Tianjin Institute of Industrial Biotechnology, Chinese Academy of Sciences, Tianjin 300308, China

[2] State Key Laboratory of Medicinal Chemical Biology and College of Pharmacy, Nankai University, Tianjin 300071, China

#Correspondence:

Qinhong Wang, Tianjin Institute of Industrial Biotechnology, CAS, 32 XiQiDao, Tianjin Airport Economic Area, Tianjin 300308, China. Email: wang_qh@tib.cas.cn

Jianpin Lin, State Key Laboratory of Medicinal Chemical Biology and College of Pharmacy, Nankai University, 92 Weijin Road, Nankai District, Tianjin, 300071, China. Email: jianpinglin@nankai.edu.cn

This PDF file includes:

Supplementary Methods

Supplementary Table S1 and Table S2

Supplementary Figure S1 to S4

Supplementary Methods

Construction of iron force field. Based on Pang's method[1], we transferred coordination bonds into electrostatic interactions by creating six dummy atoms near the metal in the direction of coordination bonds, which represented iron's six vacant sp^3d^2 orbitals. Fe and dummy atoms were constructed into a small octahedraon-shaped structure to replace Fe(III). Every dummy atom (Dz) had half a positive charge but no volume in order to share iron's charges. It would ligate with catechol, water or residues instead of Fe in terms of electrostatic energies. The distance between Fe and Dz (dummy atom) was set to 0.8 Å. As no van der Waals parameters of Fe(III) could be found, we consulted parameters of heme Fe(II) as approximation and no charge was employed(i.e., $r^* = 1.2$ Å, $\varepsilon = 0.05$ kcal·mol^{-1} and $q = 0$). Besides, the dummy atom was assigned only with charge (i.e., $r^* = 0$, $\varepsilon = 0$ and $q = 0.5$ e). All the information of Fe-Dz structure such as coordinates, bond lengths, bond angles and dihedrals, was calculated and written into two files, fe.lib and fe.frcmod, in order to load the iron force field in AMBER.

Molecular Dynamic (MD) simulation. The crystal structure of wild type of CatA (WT) was get from the Protein Data Bank (PDB ID: 1DLT)[2]. Three corresponding mutants, L73F, P76A, L73F/P76A, and WT were prepared by using the Leap module of Amber12 with the standard Amber99SB force field and our manually creating iron force field. The force field of the substrate catechol was built by the Antechamber module of AmberTools12[3]. In Leap program, Na^+ ions were added to neutralize the protein. TIP3PBOX[4] was used to add an explicit water box and the distance between the edges of the box and the closet atoms of the complexes was set to 15 Å. Every complex was saved into prmtop files and inpcrd files.

There are four steps in minimization procedure. Every step was using the steepest descent minimization, followed by the conjugate gradient minimization with the same steps. Firstly, when the protein fixed, solvent and ions were undertook a 2000-step optimization (1000 steps of steepest descent minimization and 1000 steps of conjugate gradient minimization). Secondly, two more Fe ions (the protein is dimer) were added to be optimized under a 20000-step minimization. This procedure could let the metal ligate with catechol and hydroxide. Thirdly, bonded Fe, catechol and hydroxide were

free to bind His and Tyr to form the hexacoordinated octahedraon-shaped structure under a 20000-step minimization. At last, the whole system was optimized using 5000 steps of minimization.

After the minimization procedure, every solvated complex was equilibrated by 30 ps of heating to heat system from 0 K to 300 K, 30 ps of density equilibration and 275 ps of constant pressure equilibration. All simulations were run with a 0.5 fs time step, and gradually reduced constraints on protein and langevin dynamics[5] for temperature control. Then the final phase of equilibration of every system was used to run a 2-ns MD simulation. The condition is the same as equilibration procedure but with a 1 fs time step and no restraints on hydrogen bonds.

Measuring binding pocket volumes. POVME was used to calculate the volume of binding pocket[6]. To measure the pocket, a region should be defined by overlapping spheres and right rectangular prisms to entirely cover the active site at first. One point to note is the region couldn't be too large to beyond the solvent-exposed protein atoms. Then a single volume-grid file in the PDB format is created to record the coordinates of equispaced points that fill the defined region by the algorithm. After the volume-grid file has been generated, the algorithm can systematically delete volume-grid points near protein atoms and leave points that locate in the pocket. As each of them are equispaced and has a fixed volume, the volume of binding pocket can be computed by adding up the number of these points remaining.

Supplementary Table

Table S1 Strains and plasmids used in this study

Name	Description	Sources
Escherichia coli AB2834	*aro*E mutant	*E. coli* Genetic Stock Center
E. coli trans5a	Strain for gene cloning	Beijing Transgen Biotech
E. coli BL21(DE3)	Strain for protein overexpression	Lab collection
E. coli WZI	*E. coli* AB2834 harboring pKD8.243 and pKD8.292	This study
E. coli WZK	*E. coli* AB2834 harboring pKD8.243 and pKD8.292K	This study
E. coli WZC	*E. coli* AB2834 harboring pKD8.243 and pKD8.292C	This study
E. coli WZT	*E. coli* AB2834 harboring pKD8.243 and pKD8.292T	This study
E. coli WZPL25 (WT)	*E. coli* AB2834 harboring pKD8.243 and pKD8.292PL25	This study
E. coli WZPL25G72P (G72P)	*E. coli* AB2834 harboring pKD8.243 and pKD8.292PL25-G72P	This study
E. coli WZPL25G72A (G72A)	*E. coli* AB2834 harboring pKD8.243 and pKD8.292PL25-G72A	This study
E. coli WZPL25G72V (G72V)	*E. coli* AB2834 harboring pKD8.243 and pKD8.292PL25-G72V	This study
E. coli WZPL25L73F (L73F)	*E. coli* AB2834 harboring pKD8.243 and pKD8.292PL25-L73F	This study
E. coli WZPL25L73M (L73M)	*E. coli* AB2834 harboring pKD8.243 and pKD8.292PL25-L73M	This study
E. coli WZPL25P76A (P76A)	*E. coli* AB2834 harboring pKD8.243 and pKD8.292PL25-P76A	This study
E. coli WZPL25P76G (P76G)	*E. coli* AB2834 harboring pKD8.243 and pKD8.292PL25-	This study

	P76G	
E. coli WZPL25P76V (P76V)	*E. coli* AB2834 harboring pKD8.243 and pKD8.292PL25-P76V	This study
E. coli WZPL25L73F/P76A (L73F/P76A)	*E. coli* AB2834 harboring pKD8.243 and pKD8.292PL25-L73F/P76A	This study
E. coli CatAWT	*E. coli* BL21(DE3) harboring pET-CatAWT	This study
E. coli CatAL73F	*E. coli* BL21(DE3) harboring pET-CatAL73F	This study
E. coli CatAP76A	*E. coli* BL21(DE3) harboring pET-CatAP76A	This study
E. coli CatAL73F/P76A	*E. coli* BL21(DE3) harboring pET-CatAL73F/P76A	This study
pKD8.243	*aroZ*[a], *aroY*[b]	US5487987
pKD8.292	*catA*[c], lac promoter	US5487987
pACYCP177	[d]Kan^R	New England Biolabs
pACYCP184	Cm^R, Tc^R	New England Biolabs
pET30a(+)	Kan^R, expression vector	Novagen
pKD8.292K	With the promoter of Kan^R cassette to replace lac promoter of pKD8.292	This study
pKD8.292C	With the promoter of Cm^R cassette to replace lac promoter of pKD8.292	This study
pKD8.292T	With the promoter of Tc^R cassette to replace lac promoter of pKD8.292	This study
pKD8.292PL25	With the synthetic promoter of PL25 to replace lac promoter of pKD8.292	This study
pKD8.292PL25-G72P	The derivative of pKD8.9292PL25 with mutation G72P in CatA	This study
pKD8.292PL25-G72A	The derivative of pKD8.9292PL25 with mutation G72A in CatA	This study
pKD8.292PL25-G72V	The derivative of pKD8.9292PL25 with mutation	This study

	G72V in CatA	
pKD8.292PL25-L73F	The derivative of pKD8.9292PL25 with mutation L73F in CatA	This study
pKD8.292PL25-L73M	The derivative of pKD8.9292PL25 with mutation L73M in CatA	This study
pKD8.292PL25-P76A	The derivative of pKD8.9292PL25 with mutation P76A in CatA	This study
pKD8.292PL25-P76G	The derivative of pKD8.9292PL25 with mutation P76G in CatA	This study
pKD8.292PL25-P76V	The derivative of pKD8.9292PL25 with mutation P76V in CatA	This study
pKD8.292PL25-L73F/P76A	The derivative of pKD8.9292PL25 with mutation L73F/P76A in CatA	This study
pET-CatAWT	The derivate of pET30a(+) with wild type CatA	This study
pET-CatAL73F	The derivate of pET30a(+) with mutated CatA at L73F	This study
pET-CatAP76A	The derivate of pET30a(+) with mutated CatA at P76A	This study
pET-CatAL73F/P76A	The derivate of pET30a(+) with mutated CatA at L73F/P76A	This study

Abbreviations: a, *aroZ*, the gene for 3-dehydroshikimate dehydratase. b, *aroY*, the gene for protocatechuate decarboxylase. c, *catA*, the gene for catechol 1, 2-dioxygenase. d, Kan, kanamycin; Cm, chloramphenicol; Tc, Tetracycline; R, resistance.

Table S2 Primers used in this study

Primer	Sequence(5’-3’)
Pkan-EcoRI-5	CACTGT*GAATTC*TTTTGAACTTTTGCTTTGCCACG
Pkan-3	GAATATTTTAACTTCCATGGCTGGCTCATAACACCCCTTG
Pcm-EcoRI-5	CACTGT*GAATTC*TACCTGTGACGGAAGATCACTTCG
Pcm-3	GAATATTTTAACTTCCATGGCCCATTTTAGCTTCCTTAGCTCC
Ptc-EcoRI-5	CACTGT*GAATTC*AAAACGATCTCAAGAAGATC
Ptc-3	GAATATTTTAACTTCCATGGCCGGTGCCTGACTGCGTTAG
CatA-kan-5	CAAGGGGTGTTATGAGCCAGCCATGGAAGTTAAAATATTC
CatA-cm-5	GGAGCTAAGGAAGCTAAAATGGGCCATGGAAGTTAAAATATTC
CatA-tc-5	CTAACGCAGTCAGGCACCGGCCATGGAAGTTAAAATATTC
CatA-EcoRI-3	CACTGA*GAATTC*GAACCATTTTGGTGTATTACAC
G72P	CCGTTACTCTCGCCAGGCTTGGGTTT<br>AGCTTCTTGATTGGCACCTAGCTGA
G72A	GCGTTACTCTCGCCAGGCTTGGGTTT<br>AGCTTCTTGATTGGCACCTAGCTGA
G72V	GTGTTACTCTCGCCAGGCTTGGGTTT<br>AGCTTCTTGATTGGCACCTAGCTGA
L73F	GCTGGTTTTCTCTCGCCAGGCTTGGGTTTTGACCATTACCTCGATATGCGTATGG<br>TTCTTGATTGGCACCTAGCTGATTTAAATATGCCACACCT
L73M	GCTGGTATGCTCTCGCCAGGCTTGGGTTTTGACCATTACCTCGATATGCGTATGG<br>TTCTTGATTGGCACCTAGCTGATTTAAATATGCCACACCT
P76A	GCTGGTTTACTCTCGGCGGGCTTGGGTTTTGACCATTACCTCGATATGCGTATGG<br>TTCTTGATTGGCACCTAGCTGATTTAAATATGCCACACCT
P76G	GCTGGTTTACTCTCGGGTGGCTTGGGTTTTGACCATTACCTCGATATGCGTATGG<br>TTCTTGATTGGCACCTAGCTGATTTAAATATGCCACACCT
P76V	GCTGGTTTACTCTCGGTGGGCTTGGGTTTTGACCATTACCTCGATATGCGTATGG<br>TTCTTGATTGGCACCTAGCTGATTTAAATATGCCACACCT
L73FP76A	GCTGGTTTTCTCTCGGCGGGCTTGGGTTTTGACCATTACCTCGATATGCGTATGG<br>TTCTTGATTGGCACCTAGCTGATTTAAATATGCCACACCT

Supplementary Figure

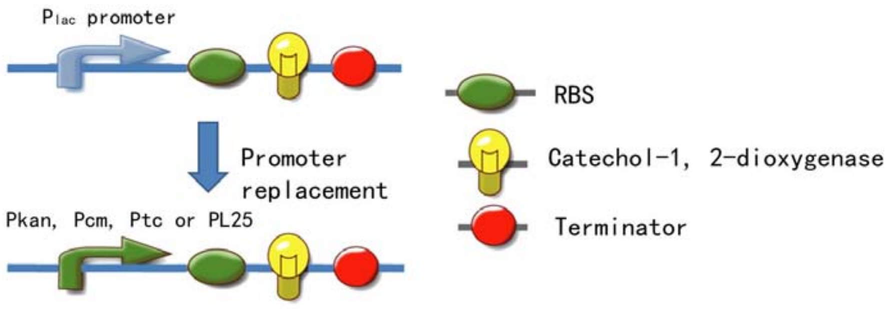

(A)

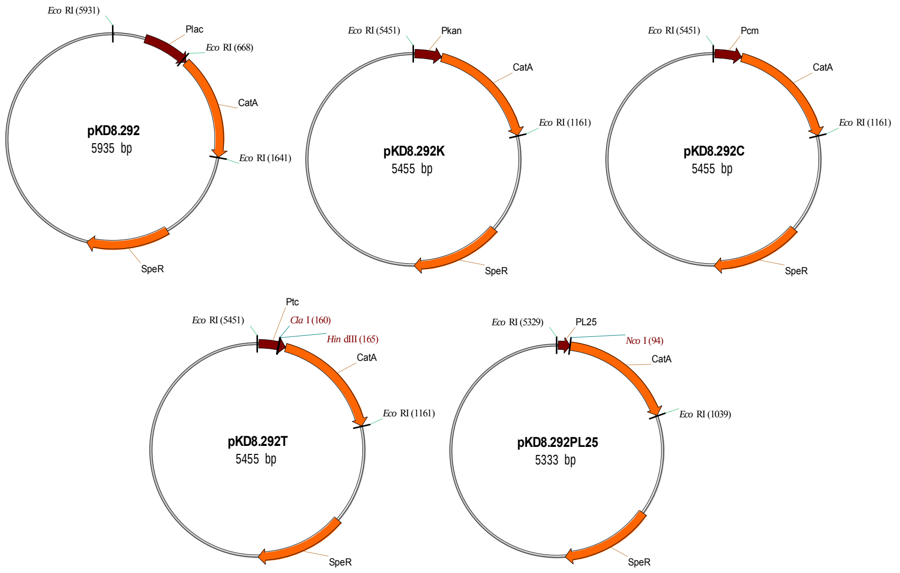

(B)

ggccagt***gaattc***tctagaatatgttatctctggcggtgttgacaacgagctggacaactggtataatgccacatgagcggataacaatttcaaggaggacagctccatg
gatggaagttaaaatattcaatactcaggatgtgcaagattttttacgtgttgcaagcggacttgagcaagaaggtggcaatccgcgtgtaaagcagatcatccatcgtgt
gctttcagatttatataaagccattgaagatttgaatatcacttcagatgaatactgggcaggtgtggcatatttaaatcagctaggtgccaatcaagaagctggtttactctc
gccaggcttgggttttgaccattacctcgatatgcgtatggatgccgaagatgccgcactaggtattgaaaatgcgacaccacgtaccattgaaggcccgctatacgtg
gcaggtgcgcctgaatcggtaggttatgcgcgcatggatgacggaagtgatccaaatggtcataccctgattctacatggcacgatctttgatgcagatggaaaaccttt
acccaatgccaaagttgaaatctggcatgccaataccaaaggcttttattcacacttcgacccaacaggcgagcagcaggcgttcaatatgcgccgtagtattattaccg
atgaaaacggtcagtatcgcgttcgtaccattttgcctgcgggttatggttgcccaccagaaggtccaacgcaacagttgctgaatcagttgggccgtcatggtaaccgc
cctgcgcacattcactattttgtttctgccgatggacaccgcaaactaactacgcaaattaatgtggctggcgatccgtacacctatgacgactttgcttatgcaacccgtg
aaggcttggtggttgatgcagtggaacacaccgatcctgaagccattaaggccaatgatgttgaaggcccattcgctgaaatggttttcgatctaaaattgacgcgtttgg
ttgatggtgtagataaccaagttgttgatcgtccacgtctagcggtgtaatac***gaattc***ccgacag

(C)

Figure S1 Reengineering *cis*, *cis*-muconic aicd synthetic pathway by constitute promoter replacement. (A) Diagram of promoter replacement. (B) The plasmid maps of pKD8.292, pKD8.292K, pKD8.292C, pKD8.292T and pKD8.292PL25. (C) The sequence of *catA* with synthetic promoter of PL25 by chemically DNA synthesis. Underlined sequence is synthetic promoter PL25. The sequence for restricted enzyme sites are displayed in ***bold italic***.

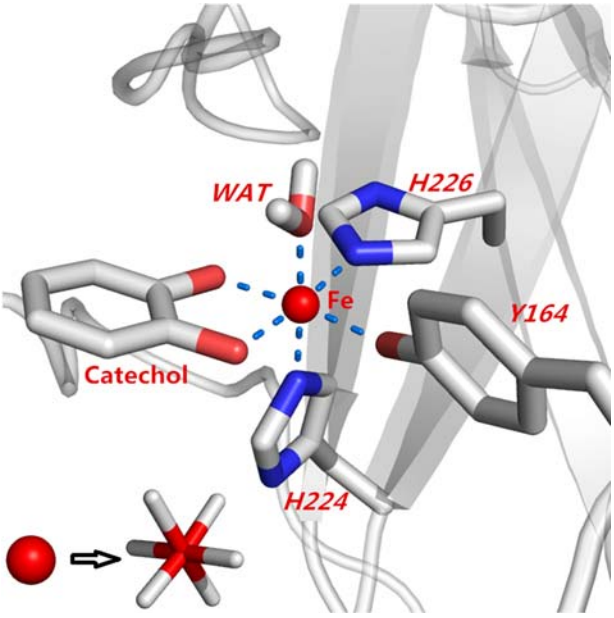

Figure S2 Molecular simulation of active site of CatA. Fe(III) has ligated five ligands: catechol, Y164, H224, H226 and a H_2O molecule (WAT). This is a hexacoordinated octahedraon-shaped structure. To run molecular dynamic simulation, Fe(III) was tackled in to a six-bonded structure by adding six dummy atoms.

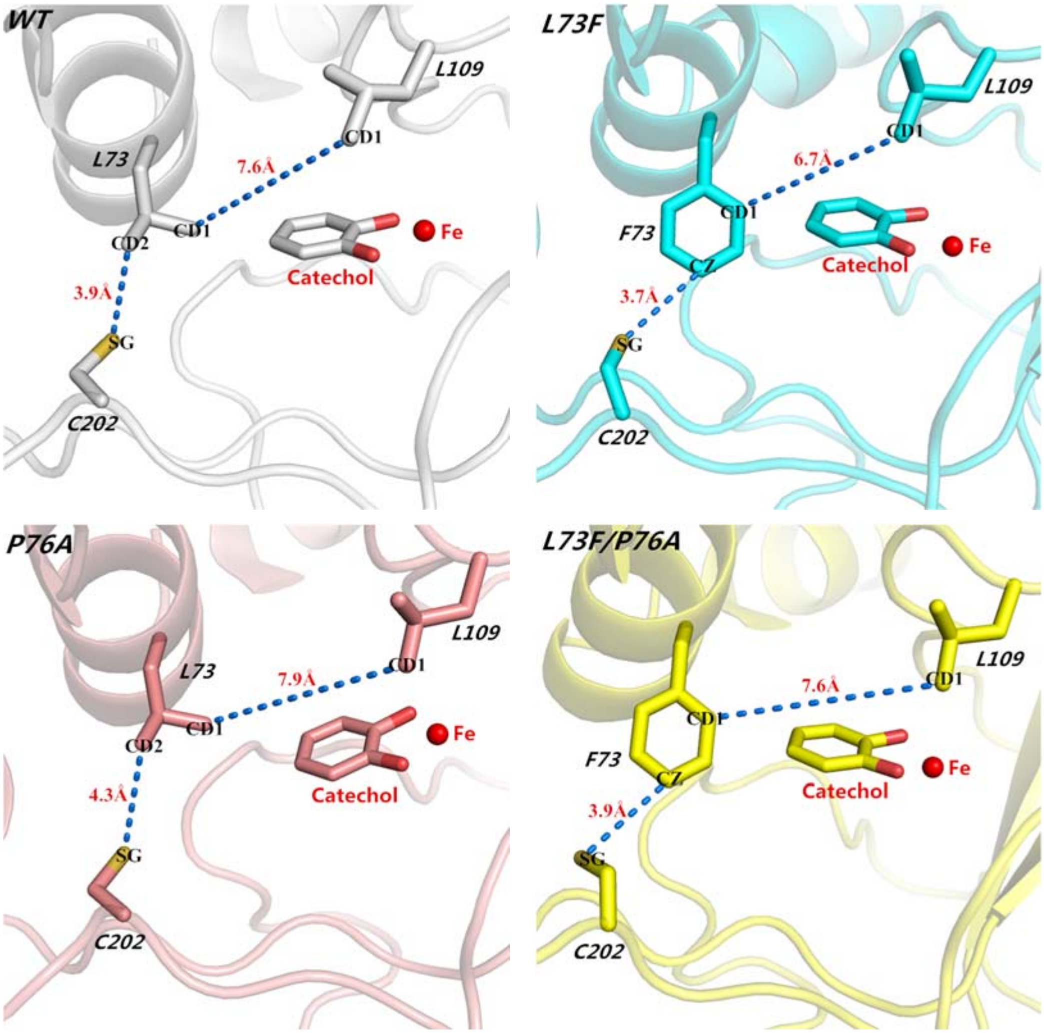

Figure S3 Distance between Leu73 or Phe73 and Leu109 and Cys202 in WT and mutants of CatA.

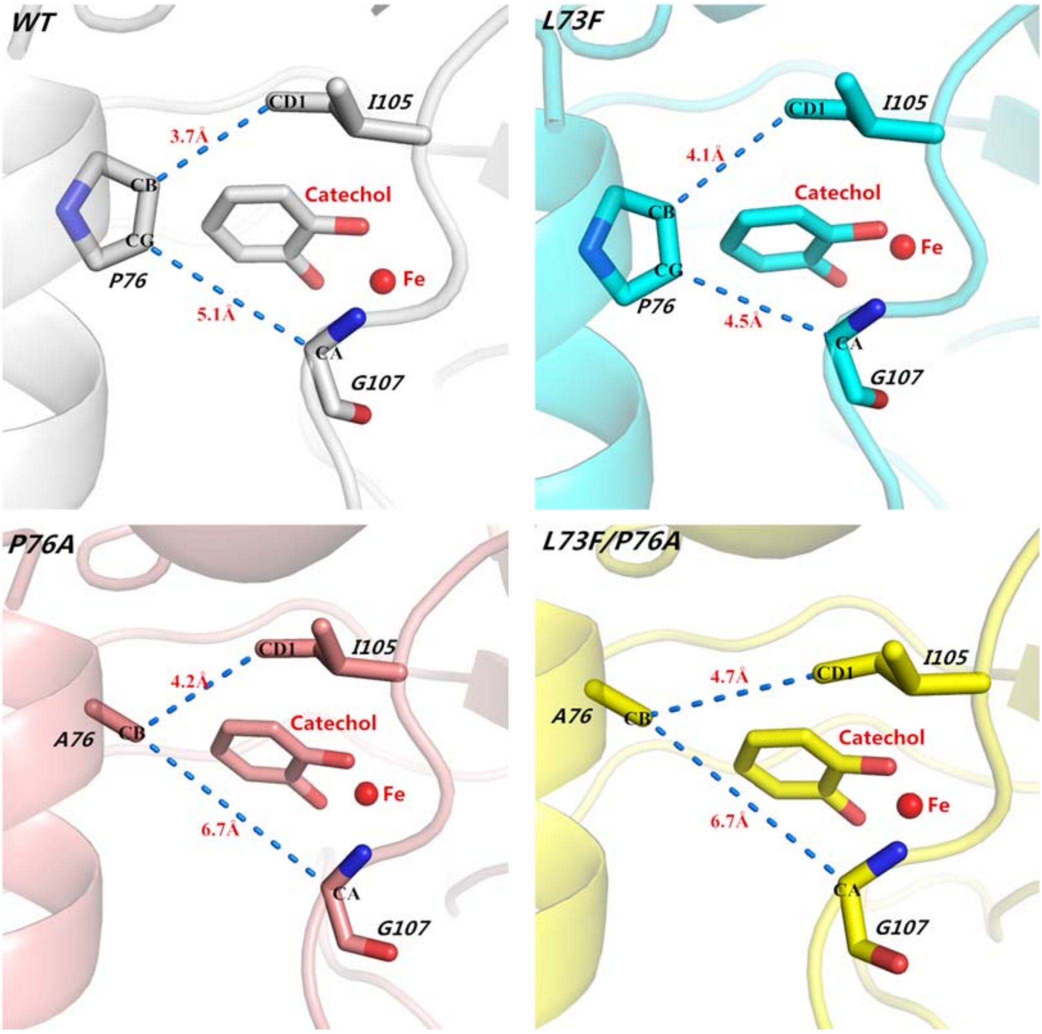

Figure S4 Distance between Pro76 or Ala76 and Ile105 and Gly107 in WT and mutants of CatA.

References

1. Pang, Y. P., 1999. Novel zinc protein molecular dynamics simulations: Steps toward antiangiogenesis for cancer treatment. J. Mol. Model. 5, 196-202.
2. Vetting, M.W., Ohlendorf, D.H., 2000. The 1.8 A crystal structure of catechol 1, 2-dioxygenase reveals a novel hydrophobic helical zipper as a subunit linker. Structure 8, 429-440.
3. Nurisso, A., Daina, A. and Walker, R.C., 2012. A practical introduction to molecular dynamics simulations: applications to homology modeling. Methods Mol. Biol. 857, 137-173.
4. Jorgensen, W.L., Chandrasekhar, J., Madura, J.D., Impey, R.W., Klein, M.L., 1983. Comparison of simple potential functions for simulating liquid water. J. Chem. Phys. 79, 926-935.
5. Pastor, R.W., Brooks, B.R., Szabo, A., 1988. An analysis of the accuracy of Langevin and molecular dynamics algorithms. Mol. Phys. 65, 1409-1419.
6. Durrant, J.D., Votapka, L., Sørensen, J., Amaro, R.E., 2014. POVME 2.0: An Enhanced Tool for Determining Pocket Shape and Volume Characteristics. J. Chem. Theory Comput. 10, 5047-5056.
